# Confound Removal and Normalization in Practice: A Neuroimaging Based Sex Prediction Case Study

**DOI:** 10.1007/978-3-030-67670-4_1

**Published:** 2021-01-30

**Authors:** Shammi More, Simon B. Eickhoff, Julian Caspers, Kaustubh R. Patil

**Affiliations:** 8 Microsoft Research, Redmond, WA USA; 9 University College Dublin, Dublin, Ireland; 10 Jožef Stefan Institute, Ljubljana, Slovenia; 11 Amazon Alexa Knowledge, Cambridge, UK; 12 Ghent University, Kotrijk, Belgium; 13 Institute of Neuroscience and Medicine (INM-7), Forschungszentrum Jülich, Jülich, Germany; 14 Institute of Systems Neuroscience, Medical Faculty, Heinrich Heine University Düsseldorf, Düsseldorf, Germany; 15 Department of Diagnostic and Interventional Radiology, University Hospital Düsseldorf, Düsseldorf, Germany

**Keywords:** Confound removal, Generalization, Interpretability, Sex classification, Neuroimaging application

## Abstract

Machine learning (ML) methods are increasingly being used to predict pathologies and biological traits using neuroimaging data. Here controlling for confounds is essential to get unbiased estimates of generalization performance and to identify the features driving predictions. However, a systematic evaluation of the advantages and disadvantages of available alternatives is lacking. This makes it difficult to compare results across studies and to build deployment quality models. Here, we evaluated two commonly used confound removal schemes–whole data confound regression (WDCR) and cross-validated confound regression (CVCR)–to understand their effectiveness and biases induced in generalization performance estimation. Additionally, we study the interaction of the confound removal schemes with Z-score normalization, a common practice in ML modelling. We applied eight combinations of confound removal schemes and normalization (pipelines) to decode sex from resting-state functional MRI (rfMRI) data while controlling for two confounds, brain size and age. We show that both schemes effectively remove linear univariate and multivariate confounding effects resulting in reduced model performance with CVCR providing better generalization estimates, i.e., closer to out-of-sample performance than WDCR. We found no effect of normalizing before or after confound removal. In the presence of dataset and confound shift, four tested confound removal procedures yielded mixed results, raising new questions. We conclude that CVCR is a better method to control for confounding effects in neuroimaging studies. We believe that our in-depth analyses shed light on choices associated with confound removal and hope that it generates more interest in this problem instrumental to numerous applications.

## Introduction

A critical challenge in applied machine learning is controlling for confounding effects as not removing them can lead to biased predictions and interpretations. This is especially true for biological data as common underlying processes introduce shared variance between the measurements, giving rise to confounding effects and blurring the boundaries between signals and confounds. Nevertheless, when confounds can be identified, removing their effects can lead to unbiased models and better understanding of the underlying biological processes.

**Fig. 1.**
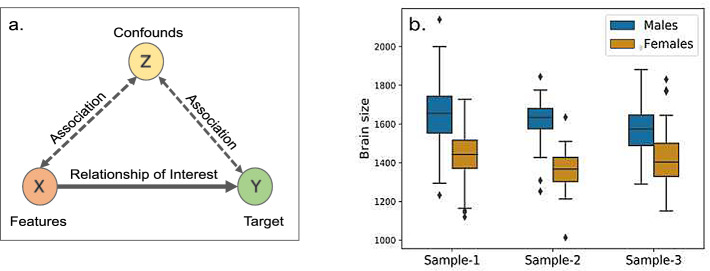
(a) Confounding effect: Confound Z influences both the features X and the target Y. In the presence of Z, the actual relationship between X and Y is masked. For sex classification, brain size is a confound (Z) as it is associated with both rfMRI features (X) and sex (Y). (b) Significant sex difference in brain size in the three data samples used in this study.

In the field of neuroimaging, predictive analysis using machine learning has gained popularity for decoding phenotypes with a clear application to understand brain organization and its relationship to behavior and disease [9, 14, 41] with a twofold aim, (1) to establish brain-phenotype relationship by estimating the generalization performance, and (2) to identify brain regions explaining the variance of the phenotype. Cross-validation (CV) is employed for the first goal while the second goal is usually achieved by identifying predictive features, e.g., features with a high weight assigned by a linear model. Specifically, in addition to information uniquely associated with the target (true signal) neuroimaging features may also contain information from nuisance sources, e.g., brain size, confounding the relationship between the neuroimaging signal and the target. In this case, both goals can yield biased results as a successful prediction might be driven by the confounding signal rather than the true signal (Fig. 1a). Thus, the confounding effects need to be removed to estimate generalizability and to gain interpretability in an unbiased way. Various alternatives exist for confound removal and are integrated within ML pipelines. However, the pros and cons of these possibilities are not well understood.

Confounding can be controlled in the experiment design phase before data collection by randomization, restriction and matching [27]. However, this is not always feasible, e.g. when all the confounds are not known. Confounds can be controlled for after data acquisition. One way is to add them as additional predictors to capture the corresponding variance. However, this approach is not suitable for predictive modelling because it is designed to control in-sample rather than out-of-sample (OOS) properties. Another method is post-hoc counterbalancing i.e., taking a subset in which there is no empirical relationship between the confound and the target [35]. Advanced techniques such as the anti-mutual information sampling [10] and stratification using pooling analysis by the Mantel-Haenszel formula [38] have been proposed. However, these methods lead to data loss and are not feasible with a small sample and a large number of confounds. Specifically, when matching sexes according to brain size, these methods will represent extremes of the population and not the whole population. Of note, confound removal can be seen as supplementary to debiasing and fair learning [2, 16, 18] but here we do not investigate this angle.

One of the most common confound control approaches while using all the data is “regressing out” their variance from the features before learning, referred to as confound regression [35] or image correction [28]. In this method, a linear regression model is fitted on each feature separately with the confounds as predictors, and the corresponding residuals are used as new “confound-removed” features. This approach can be implemented in two possible ways. The first scheme is whole data confound regression (WDCR), regresses out confounds from the entire dataset at once [28, 35, 37] followed by CV to estimate the generalization performance. WDCR has yielded inconsistent results, from a substantial drop in performance [17, 37] to a similar or slightly lower performance compared to the models without confound control [28]. This discrepancy is possibly due to differences in the strength of the relationship between the confounds, the features, and the target and implementation differences. WDCR leads to “data-leakage” as the information from the whole data is used to create the confound-removed features before CV. However, the “aggressive” confound removal by WDCR has been proposed to be desirable [25].

To alleviate issues with WDCR, a CV-consistent scheme, cross-validated confound regression (CVCR) has been proposed in which the linear confound regression models are estimated within CV using only the training subset, and applied to both the training and the validation subsets. This avoids information leaking from training into validation sets. Although both WDCR and CVCR schemes have been used in neuroimaging studies [20, 35, 45], there is a lack of information regarding how they affect the generalization estimates and interpretability with one study recommending WDCR [25] while another recommending CVCR [35].

Moreover, whether to apply a feature normalization and standardization procedures, like Z-scoring (Zero mean and unit-variance features), before confound removal or after has not been investigated. It is known that in the specific case of normalization using rank-based inverse normal transformation (INT) after confound regression may reintroduce confounding effects [24]. Such reintroduction of confounding effects can be counterproductive for model generalizability and interpretability. Furthermore, the ability of an algorithm to learn from the data might differ depending upon when normalization is applied. This lack of understanding about the interaction between confound regression and normalization makes it difficult to design ML pipelines. Lastly, building models when one suspects a shift in the covariates and/or in the relationship between the confounds, the features and the target has not been studied. Several design possibilities can be imagined and need to be evaluated.

In this work we empirically investigate three facets of the confound removal issue, (1) evaluation of the two confound removal schemes, WDCR and CVCR, for their effectiveness in removing the confounding signal and estimation of generalization performance, (2) interaction of confound removal schemes with normalization, and (3) model deployment when covariate and confounding effect shift is suspected. We consider prediction of sex from resting-state functional magnetic resonance imaging (rfMRI) data while controlling for two confounds, brain size and age. We aim to answer an important biological question “are male and female brains functionally different after controlling for the apparent difference in brain size?”. With systematic evaluation of a real-world problem reporting positive as well as negative results, we hope to attract the attention of the machine learning community to the critical problem of confound removal.

## Sex Classification and Brain Size

There are reports on differences in cognition and psychopathology between sexes [33], such as differences in spatial tasks [22], females being more vulnerable to depression [26] and autism being more prevalent in males [42]. These differences may influence diagnostic practices and help developing sex-specific treatments, making understanding neurobiology of sex differences essential. Accordingly there has been an increasing interest in finding sex differences in structural and functional properties of the brain [29, 30, 41].

Functional magnetic resonance imaging (fMRI) is a non-invasive technique used to study functional–i.e. time dependent–changes in brain activity by taking 3D MRI images in succession. Even unregulated processes in the resting brain, i.e., resting-state fMRI (rfMRI), show stable and individualized synchronies [12]. Such functional activities have been related to cognition and several phenotypes, especially using the functional connectivity (FC) (see Sect. 4.2). Based on whole-brain FC, the sex prediction accuracy of  75–80% was achieved with discriminative features mainly located in the Default mode network (DMN) [41, 45]. Another study with a lower prediction accuracy of 62% found discriminative FC in motor, sensory, and association areas [6]. Smith and colleagues [34] reported a higher prediction accuracy of 87%. A recent study reported sex prediction accuracy of 98% using multi-label learning, i.e., sex in conjunction with nine other cognitive, behavioural and demographic variables [8].

Brain size is highly correlated with sex, with larger total brain volume in males compared to females [4, 29]; and is encoded in MRI data. Figure 1b shows the difference in brain size between sexes for the data samples used in the current study. In such a scenario, even if a model decodes sex from MRI data significantly above chance, there is no clear understanding of the unique contribution of the functional features independent of brain size. It is likely that the prediction is driven partly by brain size in addition to the functional differences. Zhang and colleagues [45] have shown that the sex prediction accuracy drops from 80% to 70% after regressing out brain size from FC, indicating an apparent effect of brain size in sex prediction. Hence, there is clearly a need to study sex prediction using rfMRI while controlling for brain size.

## Experimental Setup

### Study Design

**Fig. 2.**
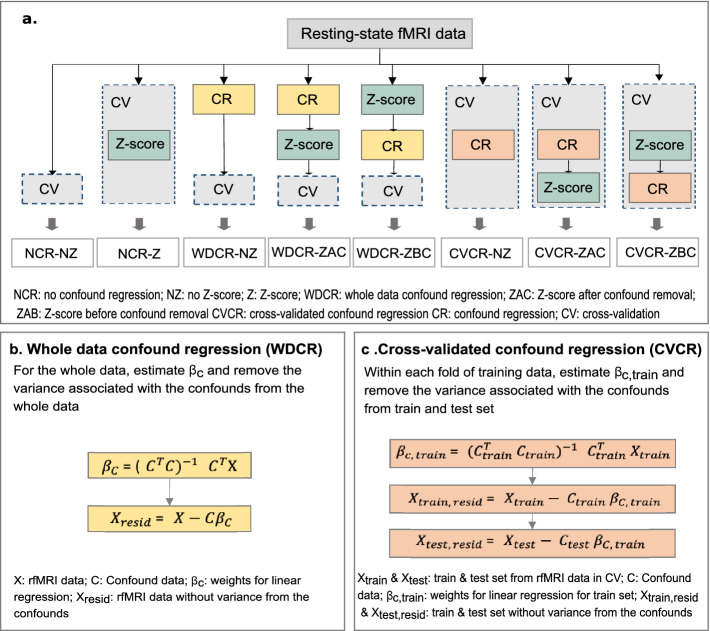
a. The schematic diagram of various combinations of confound removal schemes and Z-score for confound removal evaluated in the study. b. Whole data confound regression (WDCR). c. Cross-validated confound regression (CVCR).

With a limited and contrasting literature, there is a lack of knowledge of how to perform confound removal. Here we aimed to evaluate two confound removal schemes (WDCR and CVCR) and their interaction with the commonly used Z-score feature normalization. We evaluated eight pipelines in total (Fig. 2a);

1. No confound removal, no Z-scoring (NCR-NZ)
2. No confound removal, with Z-scoring (NCR-Z)
3. WDCR, no Z-scoring (WDCR-NZ)
4. WDCR, Z-scoring after confound removal (WDCR-ZAC)
5. WDCR, Z-scoring before confound removal (WDCR-ZBC)
6. CVCR, no Z-scoring (CVCR-NZ)
7. CVCR, Z-scoring after confound removal (CVCR-ZAC)
8. CVCR, Z-scoring before confound removal (CVCR-ZBC)

We applied these pipelines for predicting an individual’s sex using features derived from rfMRI data while controlling for two confounds brain size and age. We performed two evaluations; (1) CV to estimate the generalization performance and compared it with prediction on an OOS dataset, and (2) OOS prediction with covariate and confound shift as a model deployment scenario. The prediction performance was evaluated using AUC, F1-score and balanced accuracy.

For evaluation-1, we used a publicly available database (HCP, see Sect. 4.1) and carefully derived sample-1 (N = 377) and sample-2 (N = 54). After standard preprocessing two types of features were extracted from rfMRI data, Regional Homogeneity (ReHo) and FC (see Sect. 4.2). Each feature set was analyzed separately using Ridge Regression and Partial Least Square Regression with all eight pipelines. The generalization performance was estimated on sample-1 using 10 times repeated 10-fold CV. The OOS performance was evaluated on sample-2. By comparing the CV and OOS results, we can comment on whether the CV procedure can reliably estimate the generalization performance.

As the confounds were linearly removed from the features in a univariate way (see Sect. 3.2) multivariate confounding effects might still remain. We, therefore, assessed the effectiveness of confound removal pipelines in removing univariate and multivariate confounding effects. The Pearson correlation between each residual feature and the brain size was calculated to check for remaining univariate confounding effects. The adjusted $r^{2}$ of the multiple linear regression model predicting the brain size using residual features was used to check for remaining multivariate confounding effects.

In neuroimaging studies it is common that the data is acquired on different scanners [40] and there may exist demographic differences between samples. Such differences can lead to covariate shift [19] and by extension confound shift. An ideal model should generalize well despite such differences. To evaluate this (evaluation-2), we employed an additional sample (sample-3; N = 484) from a public dataset (eNKI, see Sect. 4.1) where demographics, scanner parameters and preprocessing are different than sample-1 and 2. We tested four ways to remove confounds from OOS data.

1. **Train-to-test**: The confound removal models from the train data were applied to the OOS data. This is the standard method.
2. **Test WDCR**: WDCR was performed on the OOS data.
3. **Test CVCR**: CVCR was performed on the OOS data, i.e. confound regression was performed within CV for OOS data and the residuals were retained.
4. **Train and test combined**: WDCR was performed on the combined train and OOS data. The data was then re-split into train and test.

Methods 2, 3 aimed to obtain confound-free OOS data, with the assumptions that confound-removed models can perform well on confound-removed OOS data as confounds are handled within a sample. Method 4 assumes that the confound removal linear models can capture variance from both train and OOS data. Note that 2, 3 and 4 can only be used with sufficiently large OOS data. WDCR models trained on sample-1 were used to predict the confound-removed OOS data. The sample-2 and sample-3 with similar and different properties to sample-1 respectively were the OOS datasets. Note that for method 1, 2 and 3 trained models (on sample-1) come from the above-mentioned pipelines used for evaluation-1.

### Confound Regression

We tested two different versions of confound regression, WDCR and CVCR (Fig. 2b and c). In WDCR, using multiple linear regression we regressed out the confounds from each of the predictors from the entire dataset before the cross-validated procedure. Note that, this procedure uses information from the whole dataset leading to data-leakage. In CVCR, we regressed the confounds in a similar way to WDCR but the confound removal models were estimated on the training data and subsequently applied to both train and validation sets. In this way, there is no leakage from train to test.

### Predictive Modelling

We used two prediction models, Ridge Regression and Partial Least Square regression. Ridge Regression (RR) uses a sum of the square penalty on the model parameters to reduces model complexity and prevent overfitting [15]. The balance between the fit and the penalty is defined using a hyper-parameter $\lambda$ which we tuned in an inner CV loop. PLS Regression (PLS) performs dimensionality reduction and learning simultaneously, making it a popular choice when there are more features than observations, and/or when there is multicollinearity among the features. It has performed well in MRI-based estimations for cognitive, behavioural and demographic variables [8, 45]. PLS searches for a set of latent vectors that performs a simultaneous decomposition of predictors and the target such that these components explain the maximum covariance between them [1]. These latent vectors are then used for prediction. The hyperparameter for the PLS is the number of latent variables which was tuned in an inner CV loop.

## Data Samples and Features

### Data Samples

This study included three samples. Sample 1 and 2 are two independent subsets of the data provided by the Human Connectome Project (HCP) S1200 release [39]. Sample-1 contained 377 subjects (age range: 22–37, mean age: 28.6 years; 182 females), sample-2 comprised 54 subjects (age range: 22–36, mean age: 28.9 years; 28 females). As the HCP data contains siblings and twins, the samples were constructed such that there were no siblings within or across the two samples, to avoid biases due to any similarity in the FC of the siblings. Within each of the two samples, males and females were matched for age, and education. Resting-state blood oxygen level-dependent (BOLD) data comprised 1200 functional volumes per subject, acquired on a Siemens Skyra 3T scanner with the following parameters: TR = 720 ms, TE = 33.1 ms, flip angle = 52$^\circ$, voxel size = 2 $\times$ 2 $\times$ 2 mm$^{3}$, FoV = 208 $\times$ 180 mm$^{2}$, matrix = 104 $\times$ 90, slices = 72. Sample-3 was obtained from the Enhanced Nathan Kline Institute–Rockland Sample (eNKI-RS) [23] with 484 subjects (age range: 6–85, mean age: 41.9 years; 311 females). Images were acquired on a Siemens TimTrio 3T scanner using BOLD contrast with the following parameters: TR = 1400 ms, TE = 30 ms, flip angle = 65$^\circ$, voxel size = 2 $\times$ 2 $\times$ 2 mm$^{3}$, slices = 64. Subjects were asked to lie with eyes open, with “relaxed” fixation on a white cross (on a dark background), think of nothing in particular, and not to fall asleep. The CAT-12 toolbox (http://www.neuro.uni-jena.de/cat/) was used to calculate the brain size of each subject based on T1-weighted images. Note the stark differences between sample-1, 2 and sample-3 in terms of demographics as well as scanner parameters. This selection was made to elucidate the common scenario of data heterogeneity.

Two-sample t-test revealed significant sex differences in the brain size across all the samples (p < 0.001; Fig. 1b). This clearly demonstrates that brain size is a confound in sex prediction. There was no difference in age between sexes in sample-1 but significant differences was observed in sample-2 and 3 (p < 0.001). Age is not expected to be related to sex but was included as a control confound.

### Pre-processing and Feature Extraction

After standard rfMRI pre-processing we extracted two types of features based on the voxel-wise time-series.

**Preprocessing.** The rfMRI data needs to be pre-processed so that the effects of motion in the scanner are removed as well as the brain of each subject is normalized to a standard brain template (e.g., MNI-152) so that they can be compared across subjects. For samples 1 and 2, the pre-processed, FIX-denoised and spatially normalized to the MNI-152 template data provided by the HCP S1200 release was used. There was no difference in the movement parameters (measured as mean framewise displacement) between males and females in both the samples. No further motion correction was performed. For sample-3, physical noise and effects of motion in the scanner were removed by using FIX (FMRIB’s ICA-based Xnoiseifier, version 1.061 as implemented in FSL 5.0.9; [13, 31]). Unique variance related to the identified artefactual independent components and 24 movement parameters [32] were then regressed from the data. The FIX-denoised data were further preprocessed using SPM8 (Wellcome Trust Centre for Neuroimaging, London) and in-house Matlab scripts for movement correction and spatial normalization to the MNI-152 template [3].

**Regions of Interest (ROI).** The Dosenbach atlas was used to extract 160 ROIs from the whole-brain data. These ROIs are spheres of 10 mm diameter, identified from a series of meta-analyses of task-related fMRI studies and broadly cover much of the cerebral cortex and cerebellum [11]. This atlas has been utilized in several brain network analyses including for sex prediction [5, 45].

**Feature Space 1: Regional Homogeneity (ReHo)** measures the similarity of the time-series of a set of voxels and thus reflects the temporal synchrony of the regional BOLD signal [44]. ReHo for each subject and each of the 160 ROIs was calculated as the Kendall’s coefficient of concordance between all the time-series of the voxels within a given ROI resulting in 160 features per subject.

**Feature Space 2: Functional Connectivity (FC)** is the correlation between the time-series of different brain regions [36]. For each subject, the time series of all the voxels within a ROI were averaged and FC was calculated as the Pearson’s correlation coefficients between them for all pairs of ROI. These were then transformed using Fisher’s Z-score. Each subject had a feature vector of length 12,720 after vectorization of the lower triangle of the 160 $\times$ 160 FC matrix.

## Results

We compiled the results from two viewpoints. We first asked which of the pipelines incorporating confound removal provides more realistic generalization performance estimates. Then we assessed the efficacy of the confound removal schemes in a model deployment scenario with data heterogeneity.

### Generalization Performance Estimates

CV is commonly used to estimate generalization performance. However, it is not without caveats [7]. Therefore, we compared CV performance of the pipelines with “true” OOS performance. In this case, the CV was performed on sample-1 and sample-2 was used as the OOS data. PLS generally performed better than RR, so in the following we focus on the PLS results.

**Table 1.**
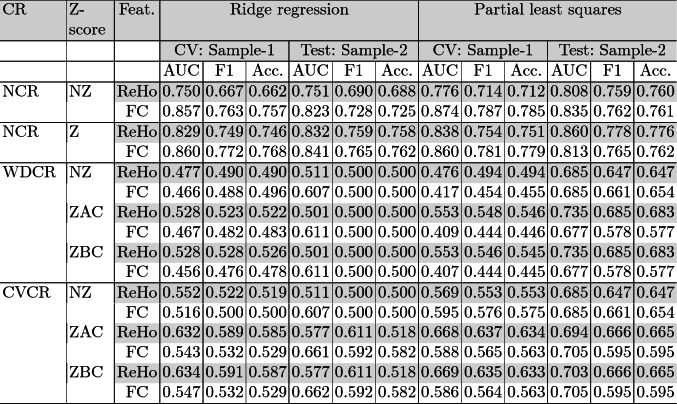
Comparison of the pipelines using RR and PLS. Models were trained on sample-1 and out-of-sample/test performance was tested on sample-2.

As expected, the CV performance was highest without controlling for confounds (Table 1). AUC and F1-scores for sex prediction with ReHo were 0.838 and 0.754 and with FC were 0.874 and 0.787, respectively. Both the schemes WDCR and CVCR showed reduced performance in line with previous studies [25, 35]. As brain size is highly correlated to sex, regressing it out from every feature can remove sex-specific information, resulting in a lower performance.

WDCR provided lower generalization estimates than CVCR, with the balanced accuracy dropping close to chance level with WDCR. One might expect higher generalization performance with WDCR as it causes data leakage from the train to the validation set violating the crucial assumption of independence in cross-validated analysis. However, in this case, it leads to worse performance. This might be because WDCR is performed on the whole dataset and hence is more aggressive in removing the confounding signal than CVCR leading to poorer performance. When the trained models were applied to OOS data, we found that OOS performance was higher than the CV estimates for most of the pipelines. This might happen if the OOS data is easier to classify. The OOS performance was closer to the generalization performance estimated with CVCR. This result suggests that CVCR is a better way to do confound removal in predictive analyses with neuroimaging data.

We then checked whether the confound removal was happening as expected. First, in a univariate way we correlated the residuals (new features) with the confounding variables. We found no significant correlation with both confound removal schemes indicating effective univariate removal of the confounding signal from the features. However, as multivariate effects might still be remaining, we used multiple linear regression to predict brain size from the residual features. With CVCR and WDCR, these models on the training sets revealed negative adjusted $r^{2}$. This indicates that there were no remaining linear multivariate confounding effects with both WDCR and CVCR. Thus the models trained with the residual features contained no information from the confounds.

These trends were similar for both ReHo and FC. Z-scoring improved the model performance with ReHo but not with FC. There was no effect of Z-scoring the features before (raw features) or after (residuals) confound removal.

**Fig. 3.**
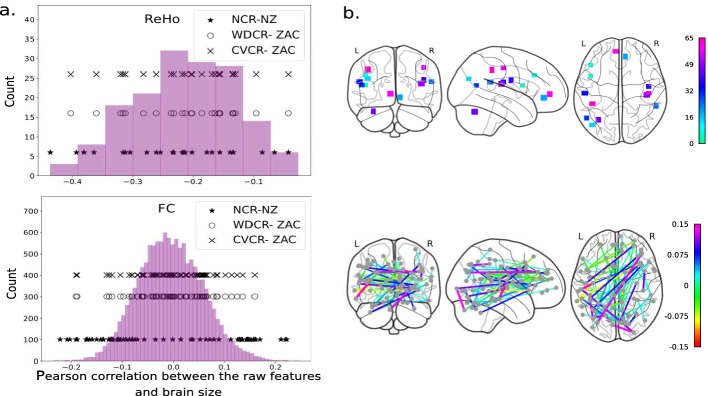
a. Pearson correlation between the raw features and the brain size as histograms. The dots show the correlations of the selected features (jittered); 25 for ReHo (top) and 70 for FC (bottom) for NCR-NZ, WDCR-ZAC and CVCR-ZAC pipelines. b. Brain regions associated with the selected features; ReHo (top, relative weights), and FC (bottom), both with the CVCR-ZAC pipeline.

### Predictive Features

One of the main objectives of a decoding analysis is to identify predictive features (brain regions) explaining the variance in phenotype. As the confounding effect can impact predictive features selection, it is important to compare them with and without confound removal. The Z-scored feature weights (the absolute value) averaged across CV runs were used to select predictive features. We found that predictive features with and without confound removal were different (Fig. 3).

We compared 25 ReHo and 70 FC features with highest absolute weights from 3 pipelines, NCR-NZ, WDCR-ZAC and CVCR-ZAC (Fig. 3a). The features selected without confound removal had relatively higher positive or negative correlation with brain size. However, after confound removal (WDCR and CVCR), for FC the features with lower correlation were selected. This suggests that the features selected after confound removal represent the functional signal predictive of sex. We then identified features selected after confound removal (CVCR-ZAC) but not selected without confound removal (NCR-NZ) (Fig. 3b). With ReHo, selected regions were in dorsolateral prefrontal cortex, inferior parietal lobule, occipital, ventromedial prefrontal cortex, precentral gyrus, post insula, parietal, temporoparietal junction and inferior cerebellum, in line with a study identifying regions in the inferior parietal lobule and precentral gyrus [43]. In contrast, another study found sex differences in right hippocampus and amygdala [21]. We found important FC features widespread across the entire brain with strong inter-hemispheric connections. In contrast to the study by Zhang and colleagues [45] we did not find many intra-network FC in the DMN. Z-score feature normalization before or after confound removal did not affect selected features.

### Out-of-Sample Performance

To study how a model deployment would work, especially in the presence of data heterogeneity common in neuroimaging studies, we tested four different ways to remove confounds from the OOS data including, applying confound models from train to OOS data using CVCR-ZAC pipeline, self-confound removal on the OOS data using WDCR and CVCR, and WDCR on the combined train and OOS data. The Z-score normalization was performed after the confound removal (ZAC) and PLS was used for prediction.

**Fig. 4.**
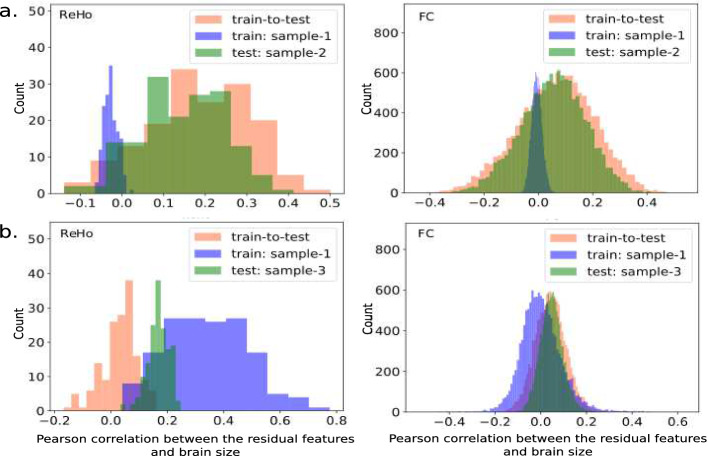
Correlation between the residual features and brain size: for out-of-sample/test data when training confound removal models were applied (orange), and for train (purple) and test (green) data when combined train and test WDCR was performed. (Color figure online)

For sample-2, train-to-test confound removal showed best performance compared to other three methods (Table 2). This is expected as the properties of these two samples are expected to be similar (i.e., no data shift). Even though, residual correlations were observed in the OOS data after applying confound models from train data (Fig. 4a), the training models were confound-free so this performance cannot be driven by confounding effects.

For sample-3 (data shift expected), we observed mixed results. For ReHo, the combined WDCR model (learned on the train data) gave highest performance (Table 2b). However, significant correlation was present between the residual features and brain size in both train and OOS data (Fig. 4b). This might indicate that the performance is driven by confounding effects. A similar model using FC was lowest performing. With combined WDCR, it seems like the dataset with higher variance dominates leaving the other part correlated, indicating it might be suboptimal. Predictions on self-confound removed OOS data (sample-3) (Test WDCR and Test CVCR) were similar to when the confound models from sample-1 were applied (Table 2a). However, the OOS performance using ReHo dropped compared to CV while that of FC improved.

**Table 2.**
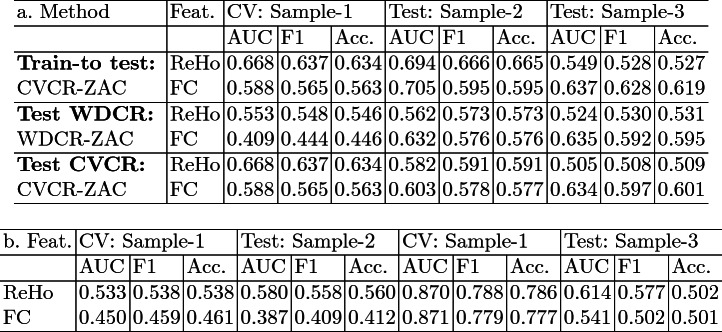
Comparison of confound removal schemes on out-of-sample/test data. a. Confound models learned from the train data (sample-1) applied to test data (sample-2 and 3), WDCR and CVCR performed only on test data. b. WDCR on the combined train and test data.

Taken together, we found that train-to-test application of confound removal models and self-confound removal to be better strategies but inconsistent across feature spaces. This raises questions regarding optimal confound removal strategies when data heterogeneity is present. Based on the results, we also speculate that covariate and confound shift is more pronounced in ReHo compared to FC.

## Conclusion

In this study, several confound removal pipelines were tested on the task of rfMRI data based sex classification. As expected, the two confound removal schemes (WDCR and CVCR) could effectively remove the signal corresponding to confounds leading to a substantial drop in prediction performance compared to without confound removal. Analyses on the residual features after WDCR and CVCR revealed that there were no remaining univariate and multivariate confounding effects. Thus, both these confound removed models should not have confound-related information encoded. We found CVCR to be a better method compared to WDCR as CVCR estimated generalization performance was closer to OOS performance. As WDCR leads to data leakage, one might expect it to be over-optimistic. However, our results point to the opposite. This is likely due to the aggressive confound removal. Our findings provide further corroboration to the idea of applying data analysis operations within the CV loop. In this work we focused on the sex prediction problem and whether our results apply to other problems remains to be seen.

The Z-score normalization of the features before or after confound removal did not affect model performance. We recommend to normalize after confound removal, as some learning algorithms might benefit from well-scaled features.

We also found that the OOS performance was best when the confound models from the train data were used, provided that the sample properties between train and test are similar but results were inconsistent with data shift. Although we used multiple regression to test for remaining multivariate confounding effects, we are not aware of a method that can directly remove multivariate effects. This calls for further investigations and development of new methods.
